# Stem cell-based approaches for glaucoma treatment: a mini review

**DOI:** 10.1515/biol-2025-1205

**Published:** 2025-12-30

**Authors:** Xirui Yang, Hao Guo, Siqi Wang, Shuwen Lu, Jixue Wang, Xingxing Yuan

**Affiliations:** Department of Ophthalmology, First Affiliated Hospital of Henan University of Chinese Medicine, Zhengzhou, China; Department of Medicine, First Affiliated Hospital of Henan University of Chinese Medicine, Zhengzhou, China; Department of Peripheral Vascular Medicine, First Affiliated Hospital of Henan University of Chinese Medicine, Zhengzhou, China; Department of Medicine, Heilongjiang Academy of Traditional Chinese Medicine, Harbin, China

**Keywords:** glaucoma, stem cells, retinal ganglion cell regeneration, neuroprotection, intraocular pressure regulation

## Abstract

Glaucoma, a leading cause of irreversible blindness worldwide, is a progressive optic neuropathy characterized by the apoptotic loss of retinal ganglion cells (RGCs) and elevated intraocular pressure. Current intraocular pressure-lowering therapies often fail to halt disease progression, creating an urgent need for neuroprotective and regenerative strategies. Stem cell therapy, leveraging the dual capabilities of differentiation and paracrine signaling, has emerged as a transformative approach for glaucomatous optic neuropathy. This review critically appraises recent advancements in stem cell-based interventions, focusing on three core therapeutic strategies: RGC regeneration, paracrine-mediated neuroprotection, and restoration of trabecular meshwork function for intraocular pressure regulation. We systematically synthesized evidence from preclinical and clinical studies, highlighting the efficacy of embryonic stem cells (ESCs), induced pluripotent stem cells (iPSCs), and adult stem cells in promoting retinal repair and neuroprotection. Despite promising results, significant translational challenges persist, including poor graft integration, tumorigenic risks, immune rejection, and the limitations of current animal models. We further discuss emerging technologies such as CRISPR/Cas9 gene editing and 3D bioprinting, which offer potential solutions for personalized and combinatory therapies. This review underscores that while stem cell therapy holds immense potential, overcoming these scientific and technical barriers is essential for its clinical translation into effective treatments for glaucoma.

## 1 Introduction

Glaucoma is a progressive optic neuropathy characterized by damage to the optic nerve and corresponding visual field loss, frequently associated with elevated intraocular pressure [1], [2], [3]. The primary mechanism of vision loss in glaucoma involves the apoptosis of retinal ganglion cells (RGCs), leading to thinning of the retinal nerve fiber layer and atrophy of retinal and lateral geniculate nucleus neurons [4]. As one of the leading causes of irreversible blindness globally, this condition stems from a complex interplay of genetic, environmental, and physiological factors. Epidemiological studies indicate that glaucoma affects approximately 3 % of individuals over the age of 40, with prevalence increasing significantly in older populations [5]. The most common forms of glaucoma include primary open-angle glaucoma (POAG) and angle-closure glaucoma (ACG). POAG, the most prevalent type, is marked by a gradual loss of vision due to progressive optic nerve damage, often linked to elevated intraocular pressure. In contrast, ACG is characterized by a sudden increase in intraocular pressure resulting from the closure of the anterior chamber angle, which can lead to acute vision loss if not treated promptly [6]. Current treatments for glaucoma focus on reducing intraocular pressure through pharmacological agents, laser therapy, or surgical interventions. However, despite these strategies, many patients continue to experience disease progression and irreversible vision loss [7].

Stem cells are unique biological entities distinguished by their ability to self-renew and differentiate into a wide range of specialized cell types. These characteristics have positioned them as a cornerstone of regenerative medicine and tissue repair [8], 9]. Stem cells are broadly classified into three main categories: embryonic stem cells (ESCs), induced pluripotent stem cells (iPSCs), and adult stem cells. ESCs, derived from early-stage embryos, exhibit pluripotency, enabling them to differentiate into virtually any cell type in the body. iPSCs, generated by the reprogramming of somatic cells, share similar pluripotent capabilities while circumventing the ethical concerns associated with ESCs. Adult stem cells, which reside in various tissues, are typically multipotent and can differentiate into a limited range of cell types related to their tissue of origin [10]. These remarkable properties underscore their immense therapeutic potential for addressing a variety of medical conditions, including neurodegenerative diseases and eye diseases [11], 12].

In the context of glaucoma, stem cells represent a promising frontier for developing novel therapeutic strategies aimed at reversing optic nerve damage and preserving vision. The regenerative potential of stem cells is being explored in several key areas relevant to glaucoma management. One significant application is the regeneration of RGCs, which are critical for visual signal transmission and are damaged in glaucoma [13], 14]. By replacing lost or damaged RGCs, stem cells hold the potential to restore visual function. Additionally, stem cells may contribute to the modulation of intraocular pressure by promoting the regeneration of trabecular meshwork cells or other intraocular structures involved in aqueous humor drainage [15]. These innovative approaches could potentially address some of the limitations of current treatments and provide a means for restoring lost vision and improving patient outcomes in glaucoma.

**Figure 1:**
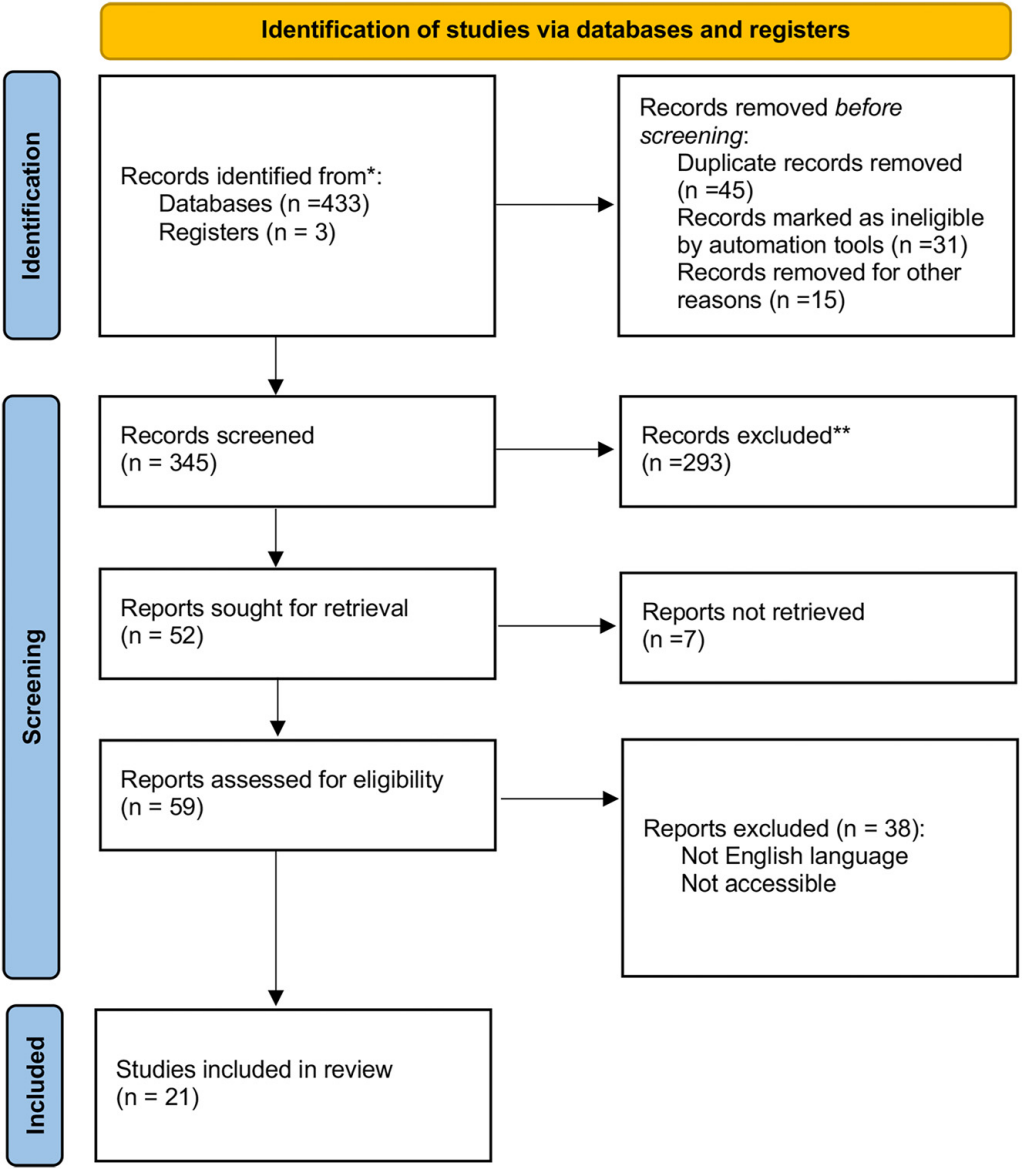
Flow diagram of literature search and study selection process.

This review aimed to summarize and critically appraise recent advancements in stem cell-based approaches for the treatment of glaucomatous optic neuropathy, highlighting both the promising preclinical evidence and the significant translational hurdles that must be overcome. We focus specifically on strategies targeting the retina, optic nerve, and aqueous humor outflow pathways, excluding anterior segment disorders unrelated to optic neuropathy. A comprehensive literature search was performed using PubMed, Web of Science, and Scopus databases, focusing on articles published primarily between 2015 and 2025. The search strategy employed combinations of key terms, including “glaucoma,” “stem cells,” “retinal ganglion cell,” “neuroprotection,” “regeneration,” “adult stem cells,” “induced pluripotent stem cells,” “embryonic stem cells,” “clinical trials,” “animal models,” and “*in vitro*.” The inclusion criteria were established to select original research articles, systematic reviews, meta-analysis, and clinical trials published between 2015 and 2025 and written in English. Studies focusing on the application of stem cells in glaucoma treatment, including mechanisms, preclinical models, and clinical outcomes, were prioritized. Exclusion criteria included reviews, case reports, editorial, conference abstracts lacking full-text availability, and non-English studies. Title and abstract were initially screened for relevance, followed by a thorough full-text assessment of selected articles. Date pertaining to stem cell types, mechanisms of action, therapeutic efficacy, challenges, and future directions were systematically extracted and synthesized. The critical appraisal focused on identifying limitations in study design, model fidelity, and the gap between preclinical promise and clinical application. Figure 1.

## 2 Applications of stem cells in glaucoma

### 2.1 Regeneration of retinal ganglion cells

The loss of RGCs is a hallmark of glaucoma and a major contributor to the progressive vision loss associated with the disease [16]. To address this, stem cell-based therapies aim to replace damaged or lost RGCs by differentiating stem cells into RGCs. Research in this field has primarily focused on ESCs, iPSCs, and neural progenitor cells. For instance, numerous studies have demonstrated the feasibility of generating RGC-like cells from these sources *in vitro*. Notably, iPSCs have been successfully differentiated into RGCs and transplanted into animal models, where they integrated into the retinal network and partially restored visual function.

Further advancing this area, Gomes et al. utilized human pluripotent stem cells (hPSCs) carrying a glaucoma-associated OPTN(E50K) mutation to explore the role of astrocytes in RGCs degeneration [17]. Their work revealed that these mutant astrocytes exhibited autophagy dysfunction and induced neurodegeneration in healthy RGCs; conversely, healthy astrocytes were able to partially rescued mutant RGCs. In another key study, microRNA-22-3p (miR-22) delivered via mesenchymal stem cell-derived small extracellular vesicles (MSC-sEVs) was shown to alleviate RGC damage in an NMDA-induced model [18]. Specifically, miR-22-sEVs thickened the retina, reduced apoptosis, and improved retinal function by inhibiting Bax and caspase-3 through the MAPK signaling pathway. Additionally, a novel protocol using CD184 and CD171 markers to derive RGC-like cells from human iPSCs yielded cells with strong proliferation and survival capabilities. These cells integrated effectively and extended into damaged optic nerves in mice, supporting their potential for cell replacement therapy in RGC degenerative diseases [19].

### 2.2 Neuroprotection and repair

Beyond mere cell replacement, stem cells confer significant potential for neuroprotection and repair within the retina. It is widely postulated that their mechanism of action primarily involves the release of neurotrophic factors and other bioactive molecules, which act to protect surviving RGCs from further degeneration [20]. This paracrine-mediated neuroprotective effect is crucial in glaucoma, where ongoing damage exacerbates vision loss. Supporting this notion, research has indicated that stem cell-derived exosomes and conditioned media alone can elicit robust neuroprotective effects in preclinical glaucoma models [21]. Moreover, beyond direct protection, stem cells can modulate the retinal immune environment to support endogenous repair processes [22].

This paracrine hypothesis is strongly supported by a growing body of evidence from specific studies. For instance, one recent study highlighted the role of microRNA-21-5p derived from iPSCs in protecting RGCs following optic nerve crush, where its secretion was upregulated in co-culture and an agonist treatment enhanced RGC survival [23]. Similarly, Ji et al. demonstrated that human adipose tissue-derived extracellular vesicles (ADSC-EVs) reduced neuroinflammation and improved RGC survival by targeting the TLR4/MAPK/NF-κB signaling pathway in a high intraocular pressure model [24]. Complementary work showed that vesicles from umbilical cord MSCs achieved similar protection in a chronic ocular hypertension model by inhibiting caspase-3 activation, underscoring a consistent anti-apoptotic role for EVs [25]. Furthermore, a comparative study revealed that the cellular origin is critical, with dental pulp-derived stem cells (DPSCs) and bone marrow-derived stem cells (BMSCs) outperforming adipose-derived stem cells (ADSCs) in providing functional and structural neuroprotection in glaucomatous eyes, positioning DPSCs as a particularly promising candidate [26].

### 2.3 Regulation of intraocular pressure

Regulating intraocular pressure remains a cornerstone of glaucoma management, and stem cells offers novel strategies to target this primary risk factor [27]. Intraocular pressure is regulated by the dynamic balance between aqueous humor production by the ciliary body and its drainage through the trabecular meshwork (TM). Emerging evidence suggests that stem cells can influence this balance through a dual approach: by regenerating TM cells to restore normal outflow function, and by potentially modulating ciliary body to reduce aqueous inflow [28], [29], [30]. This represents a paradigm shift from conventional therapies that primarily increase outflow through mechanical means or reduce inflow pharmacologically.

**Figure 2:**
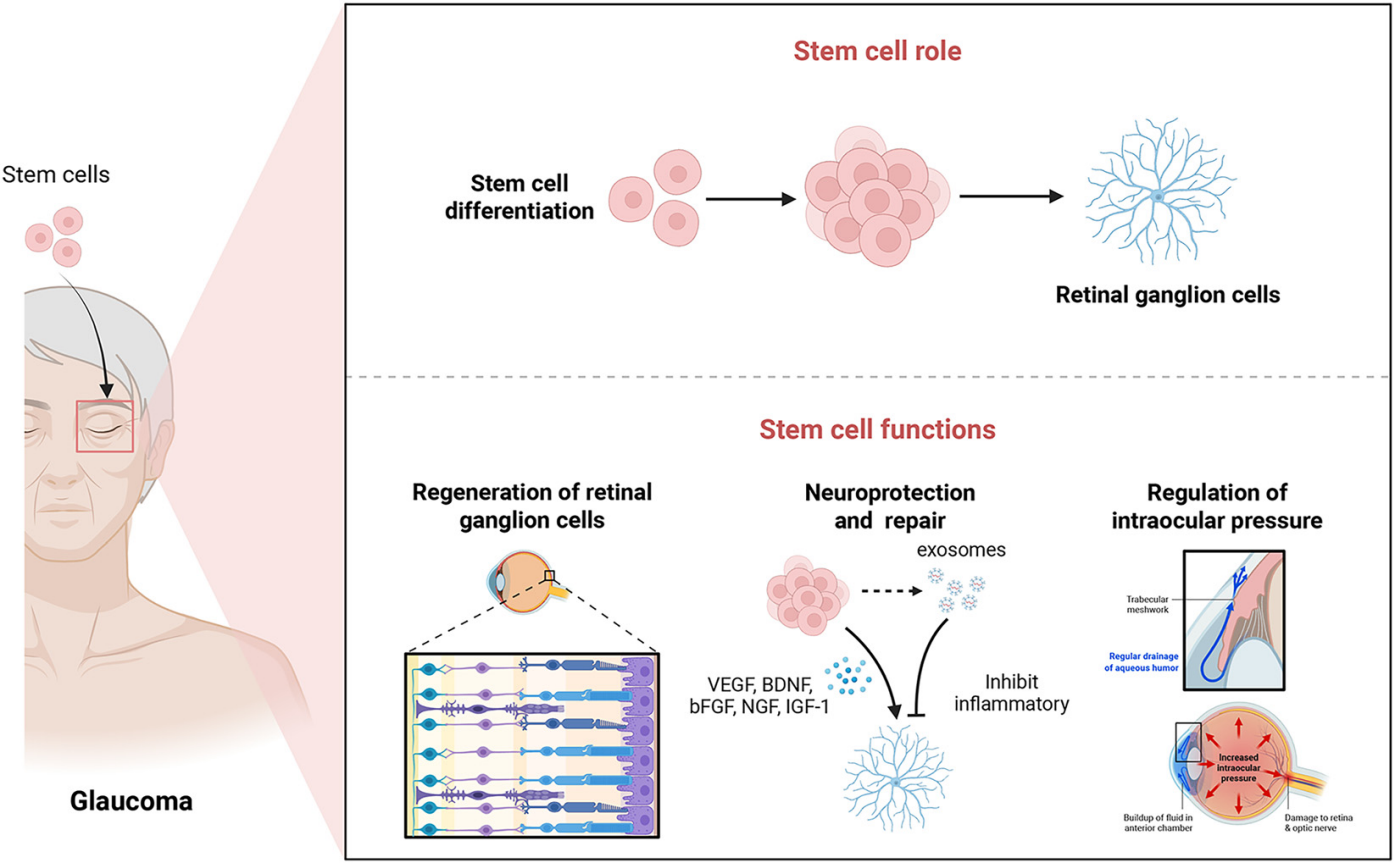
Applications of stem cells in glaucoma.

Proof-of-concept for this therapeutic approach has been demonstrated in key preclinical studies. A seminal study by Zhu et al. demonstrated that transplanting iPSC-derived TM-like cells into an ex vivo human anterior segment model stimulated the proliferation of endogenous TM cells and improved function, highlighting a potent paracrine-mediated regenerative potential [31]. In an *in vivo* setting, a study using a laser-induced TM injury model in rabbits demonstrated that a single intracameral injection of human cord blood stem cells led to a significant preservation of trabecular architecture and cellularity over 12-weeks. The integration of the injected human cells (CD34+/CD44+) along the TM beams without significant inflammation suggests a direct reparative or supportive role, offering a promising avenue for restoring physiological drainage [32]. Table 1 summarized the researches on Stem cell therapies for retinal ganglion cell regeneration, neuroprotection, and intraocular pressure regulation. Figure 2.

**Table 1:** Stem cell therapies for retinal ganglion cell regeneration, neuroprotection, and intraocular pressure regulation.

Therapeutic approach	Stem cell type	Model used	Key findings	Proposed mechanism	Reference
RGC regeneration	iPSCs	Animal models	Differentiated into RGCs, integrated into retinal network, partially restored visual function.	Cell replacement	[16]
	hPSCs	*In vitro* co-culture	Mutant astrocytes induced autophagy dysfunction and neurodegeneration in healthy RGCs.	Modeling glaucomatous pathology	[17]
	MSC-sEVs	NMDA-induced model	miR22-sEVs thickened retina, reduced apoptosis, improved function by inhibiting bax/caspase-3.	Anti-apoptotic	[18]
	hiPSCs	Mouse optic nerve crush	RGC-like cells showed strong proliferation, integrated and extended into damaged optic nerves.	Cell replacement	[19]
Neuroprotection	Stem cells	Preclinical models	Release of neurotrophic factors protects surviving RGCs from further degeneration.	Trophic support	[20]
	Stem cell exosomes	Preclinical models	Provided neuroprotective effects.	Paracrine signaling	[21]
	Stem cells	Preclinical models	Modulated retinal environment to support endogenous repair processes.	Immunomodulation & environmental modification	[22]
	iPSCs	Mouse ONC model	iPSC co-culture increased miR-21-5p secretion. Agonist treatment enhanced RGC survival via downregulation of inflammatory/apoptotic genes.	Paracrine signaling (miRNA delivery)	[23]
	ADSC-EVs	Mouse ocular hypertension	Reduced neuroinflammation, improved RGC survival, diminished microglial activation.	Anti-inflammatory (TLR4/MAPK/NF-κb pathway)	[24]
	UC-MSC-EVs	Rat chronic ocular hypertension	Reduced retinal damage, increased RGC number, inhibited caspase-3 activation	Anti-apoptotic	[25]
	ADSC, BMSC, DPSC	Rodent glaucoma	All cell types provided RGC & functional protection vs. sham. Efficacy ranking: DPSC > BMSC > ADSC.	Paracrine neuroprotection	[26].
Intraocular pressure regulation	Stem cells	Animal models	Potential to regenerate TM cells, restoring drainage and reducing intraocular pressure.	Tissue regeneration and functional restoration	[28], 29]
	Stem cells	Animal models	May affect aqueous humor production by modulating ciliary body activity.	Modulation of inflow	[30]
	iPSC-TM	Ex vivo human anterior segment	Stimulated proliferation of endogenous TM cells, improving cellularity and function.	Paracrine stimulation and tissue regeneration	[31]
	Human cord blood SCs	Rabbit laser TM injury	Preserved TM architecture & cellularity vs controls. Tracked human cells (CD34+/CD44+) integrated in TM for 12 weeks without inflammation.	Structural support and integration	[32]

## 3 Challenges and opportunities

Stem cell therapy represents a promising frontier in the treatment of glaucoma, offering potential solutions to some of the most challenging aspects of the disease. The ability of stem cells to regenerate RGCs lost due to glaucoma is particularly exciting. Early research and clinical trials suggest that stem cells can differentiate into functional RGCs, integrate into retinal structures, and potentially restore visual function [33], 34]. However, despite these promising results, several technical, scientific, and ethical challenges must be addressed.

### 3.1 Technical challenges

Efficiently guiding stem cells to differentiate into RGCs and ensuring their successful integration into the retina remains a complex task. Variability in stem cell sources, optimization of differentiation protocols, and the risk of immune rejection are significant hurdles [35]. For example, achieving high yields of specific cell types from stem cells requires refined differentiation protocols to enhance consistency and functionality [36]. Additionally, the integration of transplanted cells into the host retina is crucial for therapeutic success, as these cells must establish functional connections with existing retinal networks [37], [38], [39]. Immune rejection is another concern, as transplanted stem cells may be recognized as foreign by the patient’s immune system, potentially leading to rejection [40]. Strategies to mitigate immune responses, such as the use of immunosuppressive drugs or creating patient-specific stem cells, are under investigation.

Recent studies have highlighted the potential of BMSCs for glaucoma treatment, though initial trials involving intravitreal injection of BMSCs showed limited improvement in visual function for advanced glaucoma patients and revealed certain complications [41]. These findings underscore the need for further development of modified stem cell therapies to improve efficacy and safety.

### 3.2 Ethical and safety considerations

The use of stem cells in research and therapy raises important ethical questions, particularly regarding the source of the stem cells. For instance, the use of embryonic stem cells involves ethical debates about the moral status of human embryos. While iPSCs offer an alternative that bypasses some of these concerns, they come with their own set of ethical and practical issues [42], 43]. Ensuring that stem cell research and its applications adhere to ethical guidelines is essential to gaining public trust and advancing the field responsibly [44].

Evaluating the safety and efficacy of stem cell therapies is also crucial for their successful application in glaucoma treatment. Early clinical trials have demonstrated promising results, but comprehensive safety evaluations are necessary to identify potential adverse effects. Risks include tumor formation from undifferentiated stem cells, unforeseen immune reactions, or complications related to the transplantation procedure itself [45]. Long-term follow-up is essential to assess the durability of therapeutic effects and to monitor for delayed adverse outcomes [46].

### 3.3 Integration with existing therapies

Integrating stem cell therapy with existing treatment modalities could enhance therapeutic outcomes. Combining stem cell approaches with pharmacological treatments, laser therapy, or surgical interventions may offer a more comprehensive management strategy for glaucoma [47], 48]. Such integration could address various aspects of the disease, including intraocular pressure regulation and retinal protection. For example, combining stem cell therapy with neuroprotective drugs or minimally invasive glaucoma surgery might optimize outcomes by targeting both the structural and functional aspects of the disease [49].

## 4 Future research directions

The future of stem cell therapy in glaucoma lies in overcoming current challenges and leveraging emerging technologies to develop more effective and personalized treatments.

### 4.1 Emerging technologies

Advancements in technology hold significant promise for enhancing the efficacy and applicability of stem cell therapy in glaucoma treatment. Gene editing technologies, such as CRISPR/Cas9, offer the potential to correct genetic defects that contribute to glaucoma, enabling more precise and personalized therapeutic interventions [50], 51]. This could not only improve the quality of stem cells but also enhance their therapeutic outcomes by targeting specific molecular pathways involved in the disease.

Three-dimensional (3D) bioprinting is another exciting development that allows for the creation of complex, three-dimensional tissue structures [52], 53]. This technology could be used to generate customized retinal patches or scaffolds that facilitate better integration and functionality of transplanted stem cells. By replicating the intricate architecture of the retina, 3D bioprinting could enhance the precision and effectiveness of stem cell-based treatments [54].

### 4.2 Personalized medicine

The future of stem cell therapy in glaucoma increasingly points toward personalized medicine. Individual genetic profiles, disease mechanisms, and responses to treatments vary widely among patients. Tailoring stem cell therapies to the specific needs of each patient, based on their genetic makeup, disease stage, and other individual factors, could significantly improve treatment outcomes. Personalized approaches might involve selecting the most appropriate type of stem cells, optimizing differentiation protocols, and adjusting treatment regimens to better suit individual patient profiles [55].

For example, the discovery of a novel type of MSCs within the trabecular meshwork that may play a role in maintaining corneal health suggests new avenues for regenerative therapies for limbal stem cell deficiency [56]. This finding highlights the potential for personalized stem cell therapies tailored to specific ocular conditions.

### 4.3 Integrated therapies

Combining stem cell therapy with other treatment modalities offers a promising avenue for enhancing overall therapeutic efficacy. For instance, integrating stem cell treatments with pharmacological interventions could address multiple aspects of glaucoma simultaneously, such as neuroprotection and intraocular pressure regulation [57]. Additionally, combining stem cell therapy with laser treatments or surgical interventions might optimize outcomes by targeting both the structural and functional aspects of the disease [49]. Developing comprehensive treatment regimens that incorporate stem cell therapy with other established approaches could potentially offer more robust and durable solutions for managing glaucoma.

**Figure 3:**
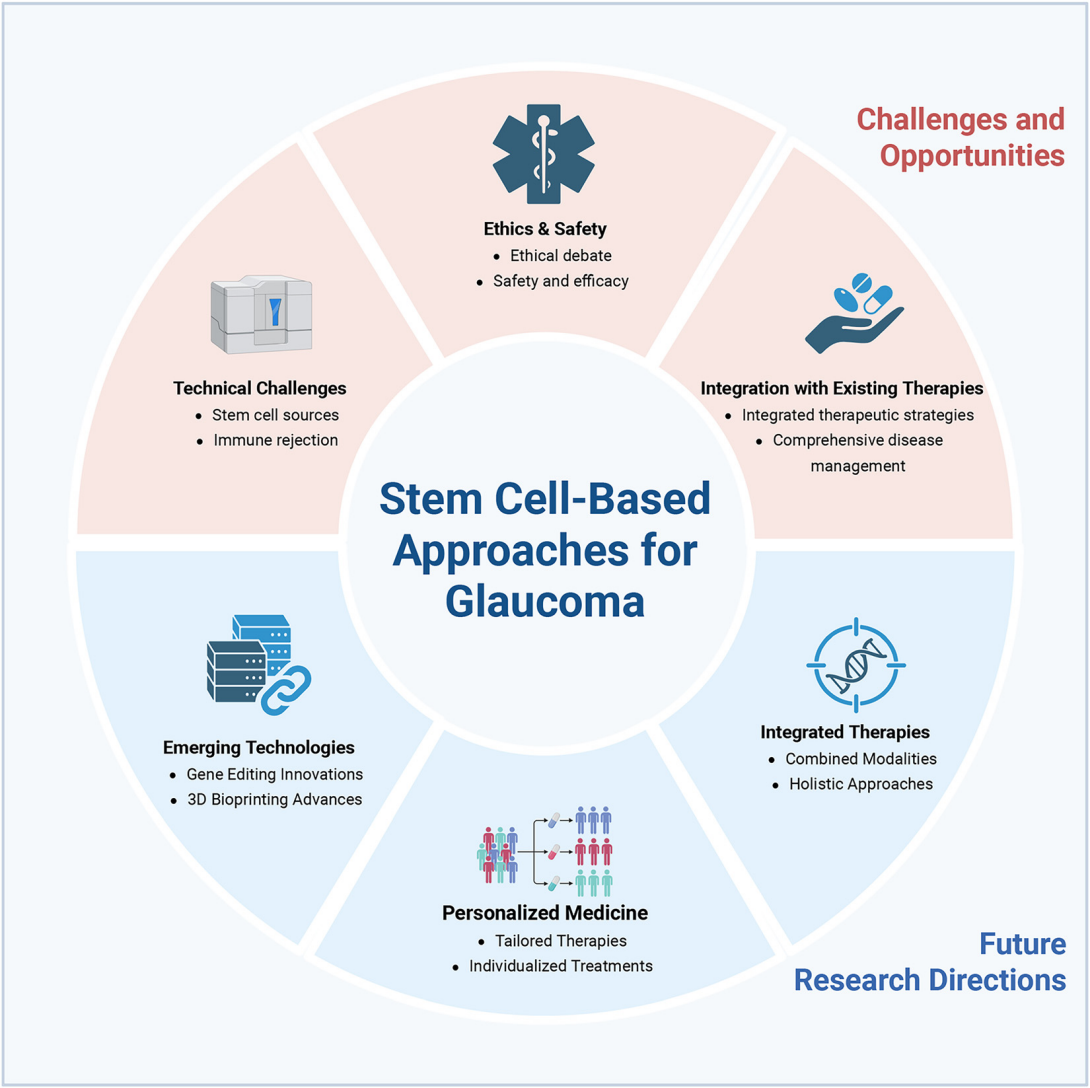
Challenges, opportunities, and future directions in stem cell therapy for glaucoma.

By leveraging new technologies, personalizing care, and combining therapies, the field of stem cell research in glaucoma has the potential to make significant strides toward more effective and individualized treatments [58]. Figure 3.

## 5 Conclusions

Stem cell therapy represents a paradigm shift in the management of glaucoma, offering the potential to not only mitigate intraocular pressure but also directly address the underlying neurodegeneration through RGC regeneration and potent paracrine-mediated neuroprotection. Preclinical studies have convincingly demonstrated the ability of iPSC, ESC, and adult stem cell derivatives to replace damaged RGCs, secrete neurotrophic factors, and restore trabecular meshwork function. However, the translation of these promising results into clinical efficacy is fraught with challenges. Key hurdles include ensuring survival, functional integration, and long-term safety of transplanted cells; standardizing differentiation protocols; and navigating ethical considerations. The future of the field lies in leveraging emerging technologies, such as CRISPR/Cas9 for gene correction in patient-specific iPSCs and 3D bioprinting for scaffold-supported cell delivery, to develop personalized and combination treatment strategies. Ultimately, overcoming these barriers will require sustained interdisciplinary collaboration, rigorous preclinical optimization in clinically relevant models, and thoughtfully designed clinical trials. By addressing these challenges, stem cell-based interventions may eventually realize their full potential to restore vision and improve the quality of life for millions affected by this debilitating disease.
